# Immune-Mediated Inflammatory Diseases: Addressing Safety Concerns and Enhancing the Accessibility of Biosimilars

**DOI:** 10.1007/s11596-026-00212-w

**Published:** 2026-06-09

**Authors:** Cláudia P. Feiteira, Isabel C. Tavares, Maria H. L. Ribeiro

**Affiliations:** 1 Faculty of Pharmacy, Universidade de Lisboa, Av. Prof. Gama Pinto, 1649-003 Lisbon, Portugal; 2 Hovione Farmaciência S.A., 2674-506 Loures, Portugal; 3 Faculty of Pharmacy, Research Institute for Medicines (I-Med.ULisboa), Universidade de Lisboa, Av. Prof. Gama Pinto, 1649-003 Lisbon, Portugal

**Keywords:** Biosimilars, Immune-mediated inflammatory diseases, Interchangeability, Nocebo effect, Biologic therapies, Healthcare accessibility, Real-world evidence

## Abstract

Immune-mediated inflammatory diseases (IMIDs), including psoriasis, lupus, multiple sclerosis, and inflammatory bowel disease, are chronic conditions characterized by immune dysregulation and excessive inflammatory cytokine expression. Biologic therapies have transformed IMID management, and the introduction of biosimilars has improved treatment accessibility by reducing costs and alleviating the burden on healthcare systems. Despite regulatory approval and demonstration of similarity to reference biologics, biosimilar adoption remains limited due to uncertainties regarding interchangeability and extrapolation, insufficient communication among stakeholders, and the nocebo effect that influences patient and physician confidence. Although regulatory agencies provide detailed guidance and European Public Assessment Reports, regulatory and perceptual barriers persist in clinical practice. This work reviews real-world and clinical trial data on switching from reference biologics to biosimilars in IMIDs and demonstrates comparable safety and efficacy outcomes. However, patient perceptions and communication strategies significantly influence treatment success. Despite the available evidence, negative perceptions and a lack of confidence persist among both patients and healthcare professionals. Promoting understanding and confidence in biosimilars can increase access and availability and, consequently, enhance the benefits associated with their wider use. Therefore, addressing educational, regulatory, and clinical practice gaps is essential for optimizing biosimilar integration in IMID management.

## Introduction

Immune-mediated inflammatory diseases (IMIDs) comprise a heterogeneous group of chronic conditions driven by dysregulated immune responses, persistent inflammation, and tissue damage. They include rheumatoid arthritis (RA), psoriasis and psoriatic arthritis (PsA), inflammatory bowel diseases (IBDs), and several overlapping systemic inflammatory disorders. Despite their diverse clinical phenotypes, IMIDs share genetic susceptibility, convergent inflammatory pathways, and frequent multimorbidity, causing significant individual and societal burdens through impaired quality of life, disability, and long-term healthcare utilization [1, 2]. Advances in genetics have reinforced the concept of shared risk architecture across IMIDs, supporting the view that common immune circuits can manifest as different organ diseases based on genetic, environmental, and stochastic factors [3, 4]. Epidemiology shows IMIDs often cluster in individuals and families, with meaningful comorbidity patterns that complicate prognosis and management [2].

The incidence of IMIDs has been increasing and can affect both men and women [2]. Therefore, it should receive the attention of stakeholders since it is a chronic condition that affects between 3% and 5% of the population with an incidence of 80 per 100,000 person-years (Table 1). Patients with one IMID are more likely to develop another. Having multiple IMIDs at the same time significantly affects patients’ quality of life and social functioning due to comorbidities like cardiovascular, metabolic, bone disorders and cognitive deficits, causing anxiety, depression, and social isolation [5, 6].

**Table 1:** Selected geoepidemiology of autoimmune diseases

Diseases	Incidence (per 100,000 person-years)	
	Europe	North America
Multiple sclerosis	0.8–8.7	2.7–7.5
Type I diabetes	> 20	10–20
Primary biliary cirrhosis	1.4–3.1	2.7 (USA)
Autoimmune hepatitis	1.07–3.0	0.5 (USA)
Graves’s disease	21–50	38
Crohn’s disease	3.1–12.7	6.9–20.0
Ulcerative colitis (UC)	4.1–16.5	8.3–19.2
Coeliac colitis	1.5–8.7 (all ages)	0.9–9.1 (all ages)
Addison’s disease	0.56–6.20	1 (USA)
Sjögren’s syndrome	5.3 (Northwest Greece)	3–5 (USA)
Systemic lupus erythematosus	1.0–5.0	1.2–8.7
Rheumatoid arthritis (RA)	9–36	31–45

Moreover, the increasing healthcare costs related to the chronicity of these diseases cause an economic burden on the healthcare system because of the need for hospitalization, frequent medical appointments, exams, and medication costs [1, 3].

Multiple environmental risk factors, such as infection and trauma, can induce this disease, but the trigger is more likely in patients who are already genetically susceptible and frequently have multiple IMIDs. Therefore, understanding the genetic evidence between the diseases and the inflammatory pathways that they share is important for the development of emerging immune therapies targeting immunopathology [1, 7].

Beyond clinical outcomes, patients describe significant practical and psychosocial impacts, emphasizing the need for coordinated, interdisciplinary care pathways that reflect the chronic, multisystem nature of these conditions [3].

IMIDs rely on complex immune networks where cytokines mediate cell communication, amplify inflammation, and coordinate effector functions. Cytokines are clinically relevant for diagnosis, stratification, prognosis, and therapy, but measuring them reliably is challenging due to low levels, limited dynamic range, biological variability, and assay differences [4]. Historical and contemporary perspectives underscore how a progressive understanding of cytokine biology has shaped modern immunology and directly translated into therapeutic innovation across IMIDs [8]. Parallel developments in single-cell technologies and multi-tissue atlases have further enabled higher-resolution mapping of immune cell states and tissue-specific inflammatory responses, enhancing efforts to identify shared mechanisms, explain heterogeneity, and refine therapeutic hypotheses across diseases traditionally considered distinct [6].

Therapeutic strategies for IMIDs have shifted from broad immunosuppression to mechanism-based interventions, notably biologic medicines that target cytokines, receptors, and immune cell trafficking. Biologic therapy has improved disease control and outcomes, reshaping treatment paradigms in rheumatology, dermatology, and gastroenterology [7]. Reviews of the therapeutic landscape describe a trajectory from early biologics to increasingly diverse targeted modalities, with ongoing efforts to optimize the long-term effectiveness, safety, and personalization of therapy [5]. However, biologics are associated with substantial direct costs, which can restrict access and may contribute to treatment delays or discontinuation, particularly in resource-limited systems. This has boosted interest in biosimilars to expand access while maintaining clinical outcomes [9].

Biosimilars are biological medicines highly similar to an authorized reference biologic, with no clinically meaningful differences in quality, safety, or efficacy. Their development follows a rigorous comparability approach, including detailed analytical tests, nonclinical assessments, and clinical evaluations as needed, in line with regulatory standards [10]. Because biologics are inherently variable and sensitive to manufacturing conditions, robust process control and adherence to international quality frameworks are essential throughout development and lifecycle management [11]. Over the past decade, the biosimilar field has matured, with stakeholders focusing on translating regulatory and scientific principles into practice—covering prescribing behaviors, pharmacovigilance, procurement, and patient communication to maximize real-world uptake and benefits [10, 12].

Switching from a reference biologic to a biosimilar or between biosimilars is key in IMID management. While trials show safety and effectiveness, implementation relies on policies, clinician and patient confidence, and shared decision-making [13, 14]. Perceived loss of efficacy or emergent adverse events may reflect the nocebo effect, needing careful communication and follow-up [15]. Success in biosimilar use depends on coordinated strategies considering evidence, regulatory standards, and health system incentives [15]. Real-world data on IBD and IMIDs helps refine monitoring and support practices [16].

Despite progress, challenges persist such as variation in uptake across countries and institutions, differing hospital procurement, and barriers related to device handling, formulation, and administration, affecting adherence and patient experience [16]. Innovations in biosimilar development and expanding therapeutic classes continue to reshape evidence and opportunities for cost savings and access [17]. Trials in IMIDs like RA and psoriasis have enhanced understanding of long-term effectiveness, immunogenicity, and patient outcomes [18]. Evidence syntheses highlight not only comparability and outcomes but also the science needed to ensure equitable access and lasting benefits across IMIDs [11, 13].

This work aims to summarize the rationale for biosimilar use in IMIDs and critically evaluate key scientific, regulatory, and clinical factors, with a particular focus on switching practices and real-world implementation. By integrating mechanistic context, regulatory principles, and emerging evidence, it supports informed decisions by clinicians, pharmacists, and stakeholders to maximize patient benefit while ensuring the safety, quality, and sustainability of IMID care [10, 12, 17, 18].

## Objectives and Methodology

This work aimed to evaluate the benefits of biosimilars, identify barriers to their use in the treatment of IMIDs, and analyze strategies to address these barriers and maximize biosimilar utilization. To achieve this objective, the following specific aims were addressed: (i) analyze regulatory strategies at the European Union (EU) and United States Food and Drug Administration (FDA) levels to promote biosimilar use in clinical practice; (ii) examine the role of the biopharmaceutical industry in the development and market availability of biosimilars; and (iii) address the barriers to the adoption of biosimilars in IMIDs and propose regulatory recommendations.

Given that this work focused on the European context, only EU-based regulatory data and guidelines, primarily from the European Medicines Agency (EMA), were considered for policy analysis. FDA-related information was included solely for comparative regulatory analysis.

A literature search strategy was performed using PubMed, the Clinical Trials Information System (CTIS), and Google Scholar for publications between 2019 and 2025. This time frame was selected to capture the most recent regulatory developments, real-world evidence, and post-approval biosimilar switching data relevant to IMIDs.

The primary search strings included: (biosimilar) AND (IMID); (immune-mediated disease) AND (biosimilar); (immune-mediated disease) AND (switching); (biosimilar) AND (switching). Additional keywords used to refine the search included: EMA, FDA, biosimilar manufacturing, approval, pharmacovigilance, and clinical practice. Boolean operators and keyword combinations were adapted to the specific requirements of each database.

Eligibility criteria included original research articles, narrative and systematic reviews, and clinical trials reporting real-world data, switching outcomes, regulatory perspectives, or implementation strategies related to biosimilars in IMIDs, as well as publications written in English.

The exclusion criteria included articles focused exclusively on oncology or non-IMID indications; studies that did not address biosimilar use, switching, or implementation in clinical practice; preclinical studies; editorials without supporting data; conference abstracts lacking sufficient methodological detail; and clinical trials without published results or relevance to the study objectives.

The initial PubMed search yielded 124 articles, 31 of which were retained after title and abstract screening. The CTIS search identified registered clinical trials; however, no trials with publicly available results relevant to this review’s objectives were included. The Google Scholar search yielded 9,880 records, reflecting its broader indexing scope and the inclusion of non-peer-reviewed materials; 10 articles were selected for review.

After screening and removing duplicates, 43 articles were included. The low yield is due to strict criteria, focus on IMID biosimilar data, and exclusion of overlapping, methodologically weak, or irrelevant articles. Many Google Scholar results were excluded for redundancy, lack of peer review, or focus on non-IMID conditions.

The selected studies collectively provided sufficient regulatory, clinical, and real-world evidence to support analyzing barriers and strategies for optimizing biosimilar use in IMIDs.

## IMIDs

IMIDs are a group of complex and chronic diseases characterized by dysregulated immune response, involving the overexpression of inflammatory cytokines. This dysregulation leads to inflammation in target organs such as joints, gut, and skin, with examples including axial spondyloarthritis (axSpA), psoriasis, PsA, IBD, Crohn’s disease, ulcerative colitis (UC), multiple sclerosis, type 1 diabetes, and systemic lupus erythematosus [1–3].

Cytokines are soluble proteins secreted by various cells, such as lymphocytes, macrophages, natural killer cells, mast cells, and stromal cells, that mediate immune or inflammatory responses. They are classified based on their cellular origin into families like tumor necrosis factors (TNFs), interleukins (ILs), lymphokines, monokines, interferons (IFNs), colony-stimulating factors (CSFs), and transforming growth factors (TGFs) [4]. Cytokines can be produced by CD4^+^ Th1 or CD4⁺ Th2 and can have a pro- or anti-inflammatory response, respectively [4, 7].

**Fig. 1:**
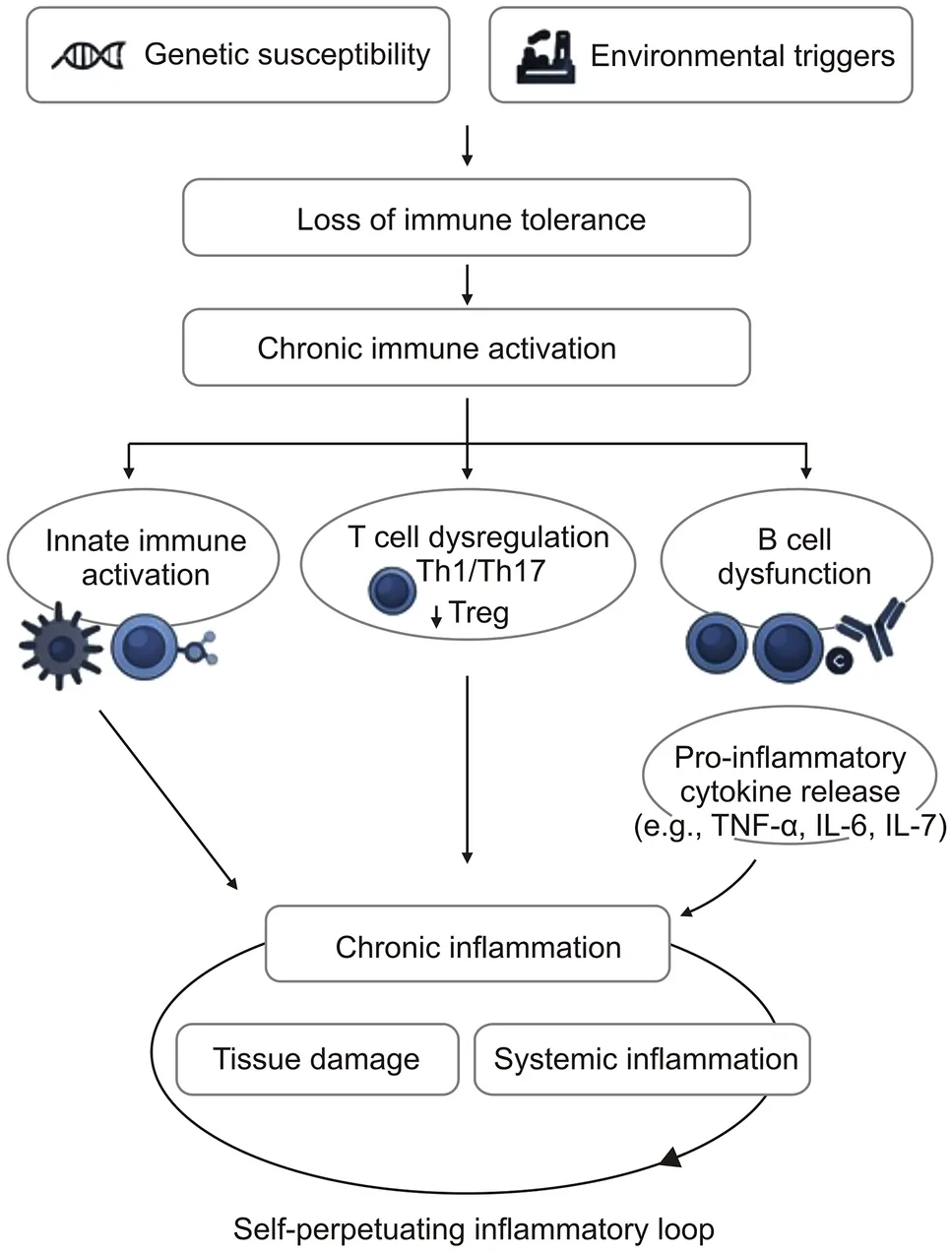
Immunopathological mechanisms common to immune-mediated inflammatory diseases (IMIDs)

Genetic susceptibility and environmental triggers lead to loss of immune tolerance, resulting in chronic immune activation (Fig. 1). This involves immune dysregulation: innate and adaptive immune responses, T-cell imbalance with increased Th1/Th17 responses and impaired regulatory T-cell (Treg) function, B-cell dysfunction, autoantibody production, and increased proinflammatory cytokine release (e.g., TNF-α, IL-6, and IL-17). These mechanisms sustain chronic inflammation, leading to tissue damage and systemic inflammatory manifestations through a self-perpetuating loop.

Proinflammatory cytokines (IL-1β, IL-8, IL-12, TNF-α, and IFNs) induce inflammatory responses and stimulate immune cells, and anti-inflammatory cytokines (IL-1, IL-4, IL-10, IL-11, IL-13, and TGF-β) inhibit inflammation and suppress immune cells. Moreover, the same cytokine can be secreted by different cells and produce different immune responses, with both pro- and anti-inflammatory effects, as with IL-6 [4]. The control of cytokine levels in serum, blood, saliva, or sweat provides important information regarding the diagnosis, stage, and prognosis of various diseases and serves as a biomarker for many immune diseases [4, 8].

IMIDs have been treated with conventional medicines as first-line therapy. However, these medicines can only control inflammation and temporarily prevent tissue damage, since they cannot target immunopathology. In contrast, biologic medicines have been revolutionary in targeting immunopathology, preventing tissue damage, and achieving long-term disease remission, markedly altering the therapeutic landscape for IMIDs since their introduction in the late 1990s and yielding substantial improvements in clinical outcomes, functional capacity, and quality of life [7]. In terms of IMIDs, there is strong evidence that biologic medicines are highly effective at inducing and maintaining disease remission. However, their high cost restricts access and places a substantial financial burden on the healthcare system [9].

Among all IMIDs, RA, psoriasis, PsA, and Crohn’s disease are the four most common IMIDs on which biologic therapy has a major impact [7].

RA involves inflammation of the synovial membrane in affected joints, triggered by antigen-presenting cells and co-stimulatory pathways that activate Th1 cells and stimulate a pro-inflammatory cytokine response, with TNF-α, IL-1, and IL-6 as key drivers. Biologics such as TNF-α inhibitors (infliximab, etanercept, and adalimumab [ADL]), anti-CD20 (rituximab), and T-cell co-stimulation blocker (abatacept) have expanded the therapeutic options and been revolutionary in treating RA [7].

Crohn’s disease manifests as a T-cell-mediated disorder that triggers an inflammatory process in the gut, with extraintestinal manifestations usually involving the skin, eyes, and joints. Infliximab and ADL are the biologics used to treat Crohn’s disease [7, 10].

Psoriasis and PsA are T-cell-mediated inflammatory conditions characterized by skin plaques and inflamed synovial tissue. The inflammatory response is triggered by the activation of CD4^+^ T-cells, which activate the pro-inflammatory cytokine response (TNF-α) and, consequently, activate CD8^+^ T-cells, which are the main effector cells. Although there is a genetic predisposition, the environment also plays an important role in psoriasis. In the case of psoriasis, conventional therapies have been ineffective at treating the disease; therefore, biologic therapies such as TNF-α inhibitors (infliximab, etanercept, and ADL) and a T-cell co-stimulation blocker (abatacept) have been revolutionary for psoriasis treatment [7].

IMIDs should be treated earlier to prevent organ damage, disease progression, and comorbidities. However, this is not common in practice due to a lack of awareness and high costs [11].

The arrival of biosimilars on the market was revolutionary for the healthcare system and for patients’ access to biologic medicines, as their lower costs introduced market competition and expanded access to these therapies. When the patent of the reference medicine expires (after 10 years of being on the market), the biosimilars can be introduced on the market, and the reference medicine can be switched to a biosimilar of the reference medicine and vice-versa, or even switched between biosimilars, which is the interchangeability concept [10, 12, 13].

Nevertheless, treatment interchangeability remains a reluctant decision for both healthcare professionals and patients. The lack of education and information to patients on biosimilar development and approval processes can lead to reluctance to switch to biosimilars and cause a nocebo effect because of their negative expectations [14]. Healthcare professionals may have concerns due to the lack of long-term data and experience regarding the outcomes of switching during biosimilar development, since they are developed under an extensive comparability exercise, especially in regard to patients on stable and long-term therapy with a reference biologic [14, 15]. Table 2 summarizes the biologic medicines used to treat IMIDs.

**Table 2:** Summary of biologic medicines, mechanisms of action, and respective disease applications (adapted from [7])

Biological medicines	Mechanisms of action	Immune-mediated inflammatory diseases (IMIDs)
Infliximab	Anti-TNF-α	RA, psoriasis, psoriatic arthritis (PsA), Crohn’s disease, UC, ankylosing spondyloarthritis
Etanercept	Anti-TNF-α	RA, psoriasis, PsA, ankylosing spondyloarthritis, juvenile idiopathic arthritis
Adalimumab (ADL)	Anti-TNF-α	RA, Psoriasis, PsA, Crohn’s disease, UC, ankylosing spondyloarthritis, juvenile idiopathic arthritis
Rituximab	Anti-CD20 monoclonal antibody (B-cell depletion)	RA, granulomatosis with polyangiitis, microscopic polyangiitis
Abatacept	Selective T-cell co-stimulation modulator (CTLA-4–Ig)	RA, PsA, juvenile idiopathic arthritis

Future IMID therapies will be based on a molecular approach using (epi)genomic, transcriptomic, proteomic, and metabolomic methods. These approaches allow prediction of the natural history of disease and the most likely adverse events, enabling selection of the most appropriate therapy for patients with IMIDs. Blood analyses and tissue-based approaches in RA, psoriasis, and IBD allow evaluation of the outcomes of future therapies [5].

**Fig. 2:**
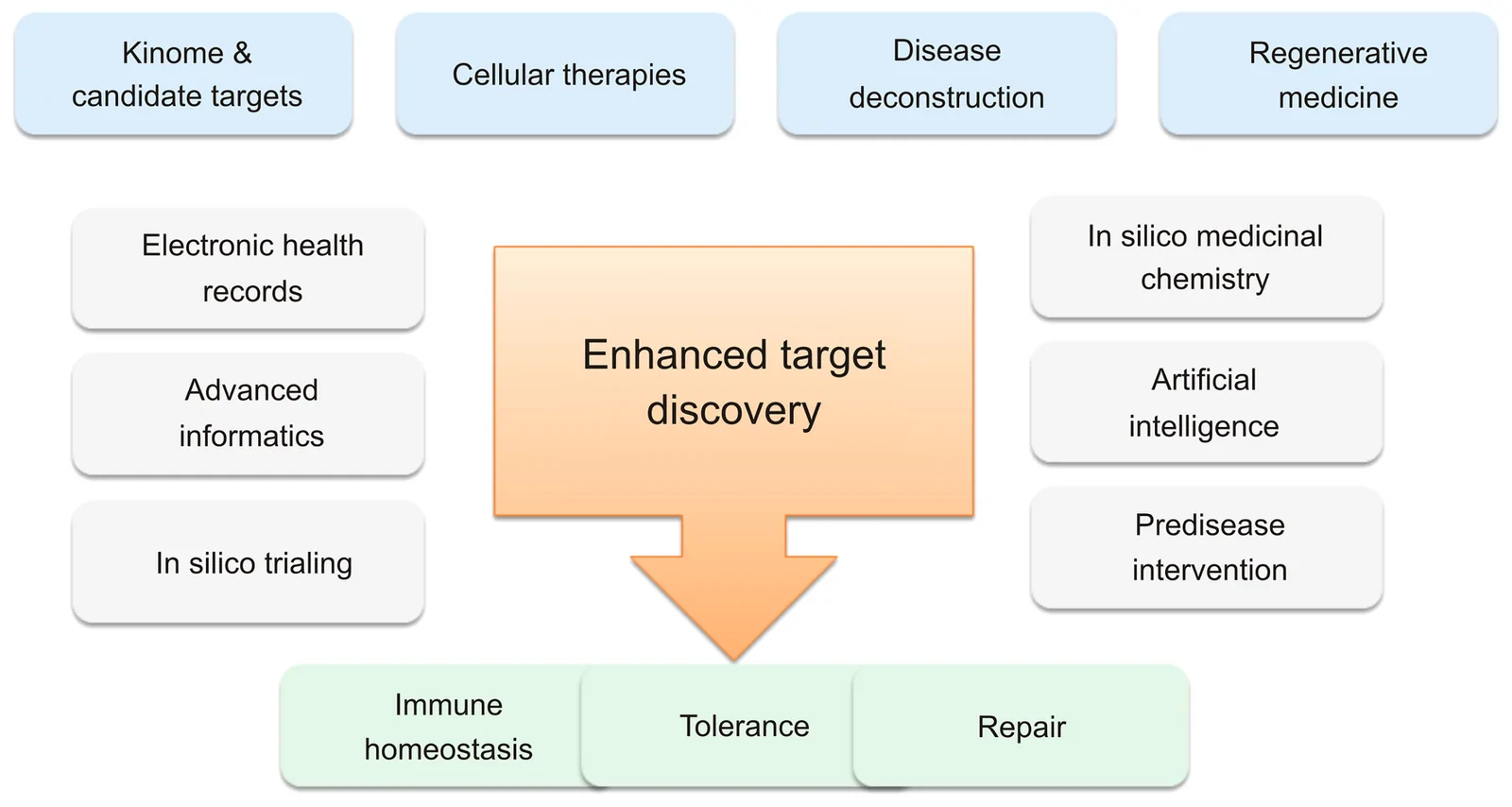
Future approaches for immune-mediated inflammatory disease therapeutics (adapted from [5])

The next phase of the molecular revolution in IMID therapeutics involves integrating strategies to identify the most precise targeted interventions (Fig. 2). These involve kinome interrogation to identify dysregulated signaling pathways, developing cellular therapies to modulate immune responses, polyomic analysis to identify patient-specific molecular drivers, and advances in regenerative medicine to restore tissue integrity and function. In parallel, the application of artificial intelligence–based approaches will accelerate and refine this molecular therapeutic approach by allowing in silico drug design, optimizing medicinal chemistry, and modeling virtual clinical trials. These advances will support the targeted selection of therapies aimed at restoring immune balance, inducing immune tolerance, and aiding in the repair of organ damage in patients with IMIDs [5].

## Development and Manufacture of Biological Medicines

Biologic medicines contain active substances derived from biological sources, such as living cells or organisms, and are produced using monoclonal antibody and recombinant DNA technologies, among others [16, 17].

The manufacture of biotechnological drug substances, including biological medicines and biosimilars, is a highly complex and tightly controlled multistep process that spans genetic engineering through the final drug product. This process includes cell line development, cell cultivation, large-scale production, product isolation, purification, formulation, and final filling and finishing operations [18].

The first phase involves genetic development of the production cell line. In this step, the gene of interest encoding the desired therapeutic protein is inserted into an expression vector, which is then introduced into a suitable host cell—commonly mammalian, bacterial, or yeast cells—to drive stable expression of the target protein. Individual cell clones that successfully express the protein of interest are selected and expanded under controlled culture conditions to establish a master cell bank (MCB), which serves as a permanent and well-characterized source of the production cells. From the MCB, aliquots are further expanded to generate a working cell bank (WCB), providing a consistent and traceable starting material for routine manufacturing runs.

Cells from the WCB are then cultivated in large-scale bioreactors under carefully optimized temperature, pH, oxygenation, and nutrient supply conditions to maximize protein production. Once the desired cell density and protein yield are achieved, the culture is harvested and subjected to downstream processing. This includes centrifugation and filtration steps to remove cells, cell debris, and bulk impurities, followed by viral clearance and elimination of host-cell proteins, DNA, endotoxins, aggregates, and metabolic byproducts.

Purification of the target protein is accomplished primarily through a series of chromatographic steps that exploit specific biochemical properties, such as size, charge, or binding affinity, thereby selectively isolating the protein of interest and removing residual contaminants. These purification and filtration steps ultimately yield a highly purified drug substance. Finally, the drug substance is formulated with appropriate excipients to ensure stability, bioactivity, and shelf life, then filled and finished into the final dosage form, making the drug product ready for clinical and commercial use [18].

Each phase is governed by specific International Council for Harmonisation (ICH) quality guidelines, including Q5 (biological products), Q6 (specifications), Q7 (good manufacturing practices for active substances), Q8 (pharmaceutical development), Q9 (quality risk management), Q10 (pharmaceutical quality systems), and Q11 (development and manufacture of drug substances), which together ensure product quality, safety, consistency, and regulatory compliance throughout the entire manufacturing lifecycle [19].

The potential Critical Quality Attributes (CQAs) of the drug substance should be identified during development and monitored throughout manufacturing to ensure properties affecting the drug’s identity, purity, biological activity, and stability [10, 20]. According to ICH Q11, “CQA is a physical, chemical, biological, or microbiological property or characteristic that should be within an appropriate limit, range, or distribution to ensure the desired product quality”. All materials in contact with the product and process parameters that affect CQAs during manufacturing development were identified and risk-assessed, and the assessments should be updated as knowledge grows [20]. Moreover, the intrinsic micro-heterogeneity of the biological material poses a challenge to maintaining quality and consistency between batches during the manufacturing process, making in-process control at critical steps essential and constant monitoring and control of CQAs necessary.

## Regulation of Biological Medicines

According to the European Medicines Agency (EMA) guidelines, a biosimilar is a “biological medicine highly similar to another biological medicine already approved in the EU (called “reference medicine”) in terms of structure, biological activity and efficacy, safety and immunogenicity profile”. Nevertheless, it is not a generic of biological medicine (reference medicine), as biological material has intrinsic microheterogeneity [17]. Owing to intrinsic changes in biologics between production batches, it is important to establish a development and approval process to address issues related to the safety and immunogenicity of biosimilars and to thoroughly understand data extrapolation across clinical indications and interchangeability.

The EMA is responsible for evaluating the majority of biosimilar applications in the EU via the centralized procedure and has one of the most rigorous regulatory frameworks for biosimilar evaluation and approval [17]. A comparison of EMA and FDA regulatory approaches to biosimilar approval is shown in Table 3. Biosimilars are evaluated using the same standards for quality, safety, and effectiveness as reference biologics, supported by comprehensive comparability exercises encompassing analytical, functional, nonclinical, and clinical data. This framework provides a robust scientific foundation for identifying biosimilarity and ensuring patient safety. All biosimilars authorized in the EU are evaluated using the same quality, safety, and efficacy standards as originator biologics, and manufacturers must comply with EU Good Manufacturing Practice (GMP) to ensure batch-to-batch consistency, traceability, and patient safety.

**Table 3:** Comparison of EMA and FDA regulatory approaches to biosimilars

Domain	EMA	FDA
Approval standard	Biosimilars are approved under the totality-of-evidence framework, which demonstrates biosimilarity to a reference biologic in quality, safety, and efficacy	Same totality-of-evidence framework for biosimilarity; additionally, a separate legal category of “interchangeable biosimilar” exists
Interchangeability	EMA considers all EU-approved biosimilars scientifically interchangeable but does not regulate switching or substitution, leaving this to national authorities	FDA designates certain biosimilars as “interchangeable,” allowing pharmacy-level substitution without prescriber intervention (subject to state law)
Switching studies	Switching is supported scientifically but not legally mandated; EMA does not require additional switching studies for approval	Switching studies are required to grant the “interchangeable” designation
Role of pharmacists	Determined by national law, automatic substitution varies widely across EU Member States	Interchangeable biosimilars may be substituted by pharmacists (depending on state regulations)
Regulatory transparency	EPARs provide detailed scientific justifications for biosimilar approval and comparability	FDA publishes review summaries and interchangeability decisions, but with less standardized clinical transparency
Main implementation challenge	Fragmented national switching policies and a lack of harmonized EU-level clinical guidance	Complexity and limited use of the interchangeability pathway; slow uptake despite legal substitution rights

**Fig. 3:**
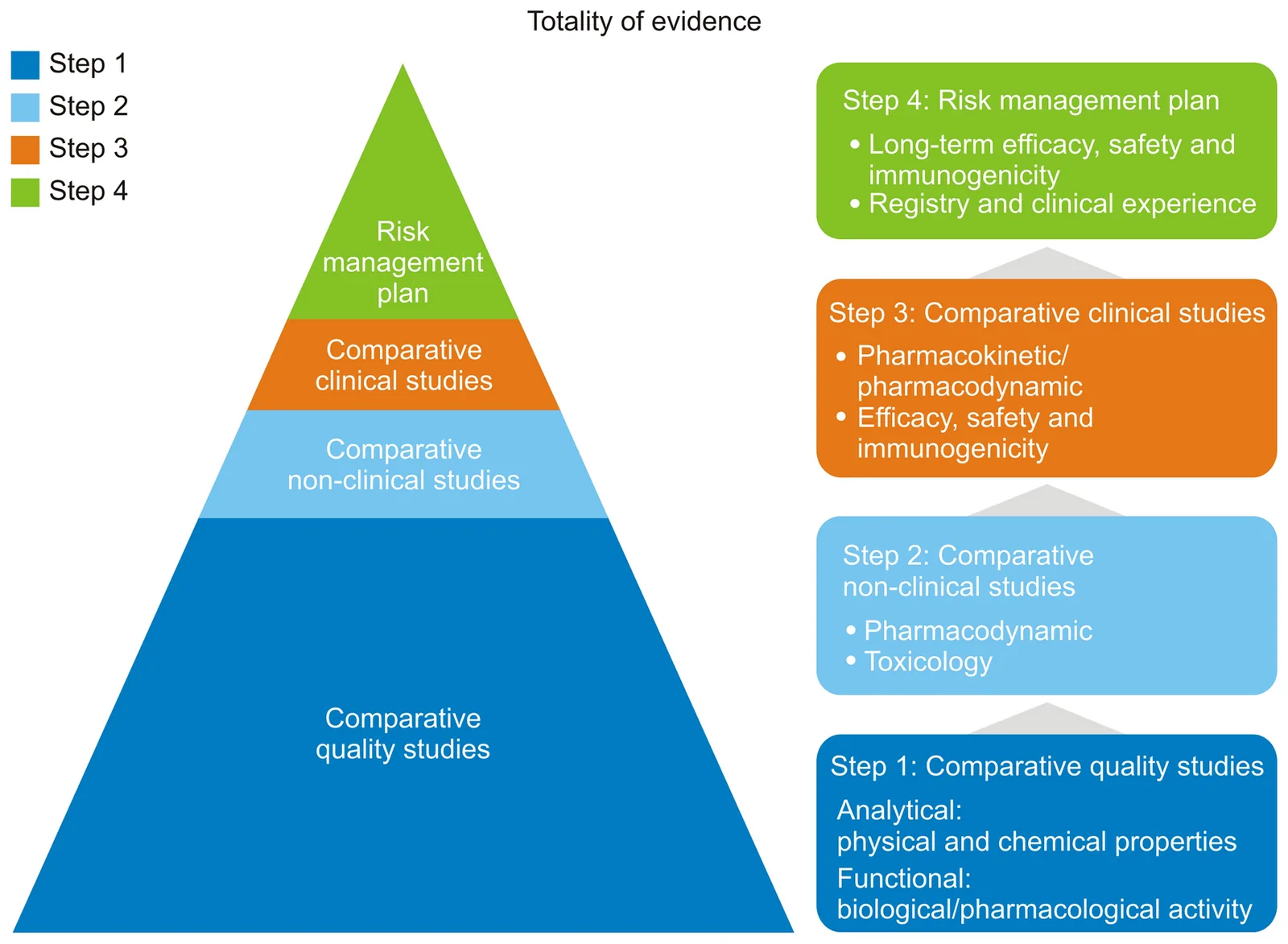
Data requirements for the approval process of biosimilars (adapted from [22])

As illustrated in Fig. 3, the biosimilar development and approval pathway is based on a comprehensive comparability exercise with the reference biologic medicine to establish biosimilarity, rather than on re-demonstrating clinical benefit. During the regulatory review process, a stepwise set of comparative studies is evaluated to confirm that the biosimilar matches the reference product in terms of quality, safety, and efficacy [14, 21].

This comparability exercise includes comparative quality studies, comprising extensive analytical and functional characterization; comparative nonclinical studies, including pharmacodynamic and toxicology assessments; and comparative clinical studies, evaluating pharmacokinetics, pharmacodynamics, efficacy, safety, and immunogenicity [17]. The aim is not to re-establish the risk–benefit profile—already shown by the reference product—but to confirm that there are no clinically meaningful differences between the biosimilar and the reference medicine [17, 21].

Once biosimilarity has been demonstrated, extrapolation of indications is possible. This means that the biosimilar may be approved for all therapeutic indications already authorized for the reference product, provided that scientific justification and the totality of evidence address potential differences in safety, efficacy, and mechanism of action across those indications. However, in certain cases, additional confirmatory clinical trials may be required for specific indications, and the justification of efficacy and safety must be robust for each approved use [17, 21]. In the EU, marketing authorization applications for biosimilars must also include a risk management plan (RMP). This plan details pharmacovigilance and risk-minimization activities to identify, monitor, and mitigate safety concerns postmarketing, ensuring patient safety. After receiving marketing approval, companies must submit a Post Authorization Safety Study (PASS) and Periodic Safety Update Reports (PSURs) that include all suspected adverse drug reactions to continuously monitor potential risks from the biosimilar and develop a strategy plan to mitigate any risks. The EMA guidance on “Biosimilars in the EU (information guide for healthcare professionals)” includes a list of biosimilars currently approved by the EMA, updated through 2019 [17].

## Role of Biosimilars in IMIDs: Economic and System-Level Impact

The emergence of biosimilars has reshaped access to biologic therapies by increasing availability (Table 4) and reducing costs. The market entry of biosimilars after the patent expiration of reference biologics promotes price competition, lowers drug acquisition costs, and mitigates the financial burden on healthcare systems [10, 12]. This economic impact is particularly important in IMIDs, where patients require long-term or lifelong biologic therapy. By lowering costs, biosimilars enable earlier treatment initiation, broader patient eligibility, and reallocation of healthcare resources to other unmet needs. There is clinical evidence that biosimilars provide safety and efficacy comparable to those of the reference medicine and that a treat-to-target approach in IMIDs using biologic therapies benefits patients and improves their quality of life.

**Table 4:** Some biosimilars approved in the EU, such as the biologic medicines, reference brands, biosimilars, and main clinical use

Class	Reference product	Reference brand name	Biosimilar	Main clinical use
Growth factors	Epoetin alfa	Eprex®/Epogen®	Binocrit	Anemia (chronic kidney disease [CKD])
			Abseamed	Anemia
	Filgrastim	Neupogen®	Zarzio	Neutropenia
			Nivestim	Neutropenia
	Pegfilgrastim	Neulasta®	Ziextenzo	Chemo-induced neutropenia
			Fulphila	Neutropenia
Hormones	Follitropin alfa	Gonal-f®	Bemfola	Infertility (assisted reproductive techniques [ART])
			Ovaleap	Infertility
	Insulin glargine	Lantus®	Abasaglar	Diabetes mellitus
			Semglee	Diabetes mellitus
	Insulin lispro	Humalog®	Insulin lispro Sanofi	Diabetes mellitus
	Somatropin	Genotropin®	Omnitrope	Growth hormone (GH) deficiency
	Teriparatide	Forsteo®	Terrosa	Osteoporosis
			Movymia	Osteoporosis
Fusion proteins	Etanercept	Enbrel®	Benepali	RA, psoriasis, PsA
			Erelzi	RA, psoriasis
Monoclonal antibodies	ADL	Humira®	Amgevita	RA, inflammatory bowel disease (IBD), psoriasis
			Imraldi	Autoimmune diseases
	Infliximab	Remicade®	Remsima	IBD, RA
			Inflectra	IBD, RA
	Rituximab	MabThera®/Rituxan®	Truxima	Lymphoma, RA
			Rixathon	Lymphoma, RA
	Bevacizumab	Avastin®	Mvasi	Solid tumors
			Zirabev	Cancer
	Trastuzumab	Herceptin®	Herzuma	HER2 + breast cancer
			Ontruzant	HER2 + cancer
Polysaccharides	Enoxaparin sodium	Clexane®/Lovenox®	Inhixa	Thrombosis prevention
			Enoxaparin Becat	Thrombosis

Therefore, biosimilars are not merely lower-cost alternatives but also enablers of a treat-to-target strategy in IMIDs, supporting sustained disease control and reducing downstream costs associated with hospitalizations, surgery, and disability. The clinical equivalence demonstrated in the respective studies directly translates into economic value, as biosimilars provide the same therapeutic outcomes at lower cost and sustainability, enabling health systems to treat more patients without compromising care quality.

## Clinical and Perceptual Barriers to Biosimilar Use

Despite the EMA’s rigorous biosimilar approval process, which relies on extensive comparability studies, there are still concerns among healthcare professionals and patients about their use. The interchangeability, extrapolation of data, and nocebo effect are the main overall issues that need to be addressed to remove/minimize barriers and maximize the use of biosimilars in IMIDs [14, 17].

**Fig. 4:**
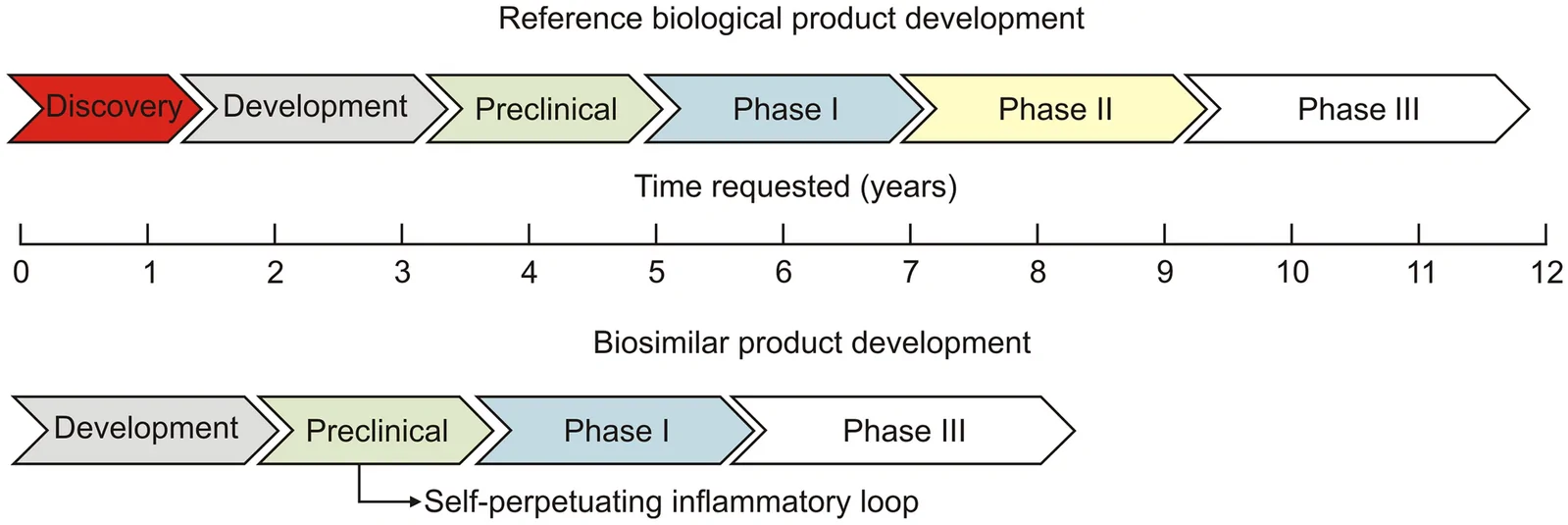
Schematic differences in the development phases of biosimilars and the reference medicine (adapted from [23])

Biosimilars are developed to demonstrate biosimilarity to the reference medicine regarding CQAs and to demonstrate equivalence in efficacy, safety, and immunogenicity. The clinical development pathway for biosimilars is more streamlined than that of the reference biologic (Fig. 4). To obtain approval, a phase I clinical study in humans is conducted to demonstrate pharmacokinetic (PK) equivalence between the biosimilar and the reference medicine. In addition, at least one phase III randomized controlled trial (RCT) must be performed in a sensitive patient population and in an indication already approved for the reference product to confirm comparable efficacy, safety, and immunogenicity. Unlike the lengthy, step-by-step process for originator biologics, biosimilar development relies on knowledge gained from the reference medicine. Because the molecular structure, mechanism of action, clinical efficacy, and safety profile of the reference product are already well established, biosimilar development focuses on demonstrating equivalence rather than re-establishing clinical benefit. As a result, biosimilars can typically be developed and approved within eight years or less, in contrast to the much longer timelines required for reference biologics (Fig. 4) [23].

This abbreviated clinical program reflects the scientific principle that once analytical, functional, and pharmacokinetic similarity has been established, only a limited confirmatory clinical study is needed to rule out clinically meaningful differences. Consequently, biosimilar development relies on a totality-of-evidence approach, in which robust analytical and PK data reduce the need for large, costly, and time-consuming Phase III trials, thereby enabling faster patient access and reduced development costs while maintaining high regulatory standards.

If efficacy and safety equivalence are demonstrated for the indication already approved in the reference medicine, extrapolation of data can be performed regarding the results obtained on the reference medicine [22]. The extrapolation of data enables the minimization of the healthcare system burden and accelerates the introduction of biosimilars on the market, as long as it is based on robust scientific evidence and demonstrates safety and biosimilarity with the reference medicine [21]. In addition, postmarketing authorization studies and real-world data enhanced the long-term effectiveness, safety, and immunogenicity of the biosimilars and increased the rigor and confidence of the approval process of biosimilars [22].

The EMA considers that a biosimilar may be interchangeable after its approval in the EU, given the robust scientific evidence and the consistent results obtained in clinical practice with biosimilars over the years [23]. However, it does not regulate how switching or substitution should be implemented in clinical practice, leaving these decisions to national authorities and healthcare systems.

Interchangeability means that during a patient’s treatment, the reference medicine can be replaced with a biosimilar, and vice versa, or a biosimilar can be replaced with another biosimilar. Interchangeability can be implemented through switching, when performed under the prescriber’s control, or through substitution, which is performed at the pharmacy level without consulting the prescriber. However, the decision on how to implement interchangeability through switching or substitution is not the responsibility of the EMA. This division of regulatory responsibilities has resulted in heterogeneous national policies on switching, substitution, and pharmacist involvement across Europe, which, in turn, contribute to inconsistent clinical implementation and persistent uncertainty among healthcare professionals and patients.

Despite the robust scientific, regulatory, and clinical evidence, biosimilar uptake in IMIDs remains suboptimal. Beyond regulatory and clinical uncertainties, behavioral and psychological factors—particularly the nocebo effect—constitute a major and underrecognized barrier to successful biosimilar switching. It refers to the occurrence or worsening of symptoms that arise from negative patient expectations rather than from pharmacological differences between treatments. In the context of biosimilar switching, this phenomenon manifests as subjective loss of efficacy, new adverse events, or treatment dissatisfaction despite objective clinical equivalence. Importantly, this effect is not a theoretical concern: multiple real-world switching studies demonstrate that discontinuation rates after nonmedical switching are frequently driven by patient-reported symptoms that cannot be explained by immunogenicity or pharmacokinetic changes [14].

In IMIDs—conditions already characterized by fluctuating symptoms, pain, fatigue, and psychological distress—patients are particularly vulnerable to expectation-driven symptom amplification. When patients perceive biosimilars as inferior, “cheaper”, or less rigorously tested, this perception can translate directly into poorer adherence, higher rates of adverse event reporting, and premature treatment discontinuation, thereby mimicking true therapeutic failure. Crucially, the nocebo effect is not driven by the biosimilar itself but by the way the switch is communicated and implemented. A lack of transparency, abrupt nonmedical switching, or inadequate explanation of biosimilar development can undermine patient trust and amplify negative expectations. Conversely, studies consistently show that patients who receive structured education and participate in shared decision-making exhibit higher persistence rates, fewer subjective adverse events, and greater acceptance of biosimilars.

Therefore, the nocebo effect represents a modifiable barrier to biosimilar success. Addressing this issue requires targeted communication strategies, patient education, and clinician engagement rather than additional pharmacological evidence. Recognizing and mitigating the nocebo effect is thus essential for fully realizing the clinical and economic benefits of biosimilars in IMIDs. The determinants of the nocebo effect in biosimilar switching are multifactorial and involve patient, physician, healthcare system, and social influences. These factors, together with their triggers and mitigation strategies, are summarized in Table 5, providing a practical framework for clinicians to prevent nocebo-driven treatment failure.

**Table 5:** Nocebo effect in biosimilar switching: risk factors, triggers, and mitigation strategies in IMIDs

Factors	Nocebo risk factors	Common triggers during biosimilar switching	Evidence-based mitigation strategies
Patient-related factors	High anxiety or depression; history of multiple treatment failures; high symptom sensitivity; low health literacy; negative beliefs about generics or biosimilars	Fear of “cheaper” medicine; concern about reduced efficacy; worry about safety or immunogenicity; prior negative drug experiences	Use validated expectation or medication sensitivity questionnaires; provide tailored education; reassure using evidence of equivalence; schedule early follow-up after switching
Physician-related factors	Lack of confidence in biosimilars; neutral or hesitant language; limited knowledge of switching data	Ambiguous or cautious phrasing when proposing a switch; inconsistent information; overemphasis on cost rather than clinical equivalence	Training in biosimilar communication; use positive framing; emphasize regulatory rigor, safety, and real-world evidence; align all staff messaging
Healthcare system factors	Non-medical switching driven by procurement; lack of continuity of care; limited consultation time	Abrupt or mandatory switching; lack of patient involvement; poor explanation of rationale	Implement shared decision-making; provide standardized educational materials; ensure time for patient questions; avoid surprise switching
Treatment-related factors	Route of administration changes; different device appearance; injection discomfort; packaging differences	New injection device; different labeling or packaging; perceived formulation changes	Hands-on training with new device; reassurance that formulation and dose are equivalent; normalization of minor injection-site reactions
Social and informational environment	Exposure to negative media; peer anecdotes of treatment failure; online misinformation	Social media stories about biosimilar “failures”; conflicting online information	Provide credible written and digital resources (EMA, EPAR); encourage discussion of concerns; use patient support groups positively
Clinical context	Stable patients fearful of losing disease control; long-term use of reference biologic	Switching during remission; fear of flare or relapse	Explain equivalence data in stable populations; present switching studies; offer monitoring and easy access to care if symptoms occur
Expectations and beliefs	Low trust in the healthcare system; belief that original brand is superior	Perception that biosimilars are inferior or experimental	Explicitly explain biosimilar approval pathway; stress EMA comparability requirements; highlight widespread real-world use

## Clinical Equivalence—Real-World Data

The treat-to-target approach in IMIDs was revolutionary in targeting immunopathology, reducing morbidity, hospitalizations, and surgical interventions, and reducing the burden on the healthcare system. Biosimilars are among the revolutionary medicines used to treat IMIDs, enabling earlier access to treatment and increasing the likelihood of achieving disease remission.

A study on patients’ access to ADL and tocilizumab revealed that biosimilar uptake after their market introduction varied across European countries due to a lack of awareness and education initiatives for healthcare providers and insufficient incentives for biosimilar use [24]. We reviewed the literature of clinical trial articles regarding the outcomes of switching to biosimilars in IMID treatment and the barriers to their use. The outcomes of the four most common IMIDs (RA, IBD, psoriasis, and PsA) were evaluated through real-world data.

### RA

Study 1 is a randomized, double-blind, phase III study that was performed to randomly distribute the patients to receive biosimilar therapy (ADL-PF) or reference medicine therapy (ADL, Humira), without knowing which treatment each patient was receiving to minimize bias. This trial aimed to assess the efficacy, safety, and immunogenicity of the biosimilar from week 52 to 92. No clinically meaningful differences in safety, efficacy, or immunogenicity were observed between patients who maintained ADL-PF for 78 weeks and those who switched to ADL-PF at week 26 or 52 (Table 6) [25]. ADL is a recombinant immunoglobulin G1 monoclonal antibody that binds specifically to TNF-α to prevent its interaction with surface TNF receptors to regulate the abnormal inflammatory response.

**Table 6:** Summary of the RA studies

Study	Description	Aim	Type of study	Outcome	References
1	Switching from ADL to biosimilar ADL-PF	Evaluate efficacy, safety and immunogenicity	Phase III, randomized, double-blind	No clinically meaningful differences	[25]
2	Switching from rituximab to a biosimilar CTP-10	Evaluate the efficacy, safety and immunogenicity	Phase III, multi-national, randomized, double-blind	No clinically meaningful differences	[26]
3	Switching from ADL to biosimilar SDZ-ADL	Evaluate the effectiveness and safety profile of treatment	Randomized, double-blinded, parallel-group, multicenter study in patients with rheumatoid arthritis	Meaningful improvements and quality of life scores were observed	[27]
4	Switching from ADL to biosimilar FKB327	Evaluate the efficacy and immunogenicity	Randomized, double-blind	No clinically meaningful differences	[28]

Study 2 aimed to evaluate the efficacy and safety of switching from the reference medicine monoclonal antibody rituximab to the biosimilar CT-P10 during a phase III, multinational, randomized, double-blind study over 72 weeks. Switching from rituximab to CT-P10 was well tolerated, and no clinical differences in efficacy, safety, or immunogenicity were observed. The long-term use of CT-P10 was demonstrated to be effective [26].

Study 3, which switched from ADL (Humira) to the biosimilar SDZ-ADL, was reviewed. This study was separated into two parts: one randomized, double-blinded, parallel-group, multicenter study in patients with RA, and another randomized, double-blinded, multicenter, phase III confirmatory study in patients with moderate‐to‐severe chronic plaque psoriasis. Meaningful improvements in patient-reported outcomes (PROs) and quality-of-life scores were observed in both the SDZ-ADL and reference-medicine treatment groups [27].

Study 4 is a randomized, double-blind study switching from ADL to the biosimilar FKB327 that was performed in 728 patients with RA and demonstrated comparable efficacy and immunogenicity profiles at week 24. Afterward, they were randomized 2:1 to remain on the same treatment or switch to the other treatment through week 54. It was demonstrated that switching or double switching between treatments did not affect treatment efficacy or immunogenicity [28].

### IBD

IBD is characterized by inflammation of the gastrointestinal tract and encompasses Crohn’s disease and UC [29]. A NOR-SWITCH trial (Study 1) was conducted in IBD patients receiving stable, long-term infliximab treatment (Table 7). The study involved 482 patients who were randomized to continue infliximab treatment or switch to an infliximab biosimilar. After 52 weeks, no significant differences or worsening of the disease were observed between the groups. At week 52, patients could switch from infliximab to an infliximab biosimilar and vice versa, or continue the treatment. After 26 weeks, no significant differences were observed, confirming the efficacy of the infliximab biosimilar [30].

**Table 7:** Summary of IBD studies

Study	Description	Aim	Type of study	Outcome	References
1	Switching from infliximab to biosimilar infliximab	Evaluate the efficacy and safety	Randomized	No clinically meaningful differences	[30]
2	Switching from ADL to ADL-adbm	Evaluate the efficacy and safety	Phase III randomized, double-blind, multicenter study	Experienced adverse events: injection-site reaction and upper respiratory tract infections	[31]
3	Switching from infliximab to CT-P13	Evaluate safety, immunogenicity and tolerability	Multicenter, parallel cohort, non-inferiority study in Australia	No clinically meaningful differences	[32]
4	Switching from ADL to SB5	To prevent the nocebo effect through educative initiatives	Phase IV, multicenter, interventional cohort study	Patients were confident to switch. Few discontinued due to injection site pain	[33]
5	Switching from ADL to SB5	Evaluate safety and effectiveness	Observational 12-month study, multicenter cohort with patients naïve to ADL or already treated	Clinically effective and presents a similar safety profile	[34]

Study 2 (VOLTAIRE-CD) was performed in patients with moderate to severe active Crohn’s disease to compare the efficacy and safety of an ADL biosimilar (ADL-adbm) with those of the reference ADL. VOLTAIRE-CD was a phase III randomized, double-blind, multicenter study, with 147 patients (90% on ADL-adbm and 94% on reference ADL) who had a clinical response at week 4. At week 24, patients who responded to treatment using the reference ADL were switched to ADL-adbm, as they experienced adverse events during weeks 24–56. The most common adverse events were injection-site reactions and upper respiratory infections, with injection-site reactions being the most frequent [31].

Study 3 was a switch from infliximab (Remicade) to the biosimilar CT-P13 (Inflectra) in Australian patients with moderate to severe IBD. A multicenter, parallel cohort, noninferiority study was performed across seven hospitals in Australia over 48 weeks, including patients who switched to the biosimilar and those who maintained the reference medicine. It was concluded that switching to biosimilar infliximab was safe and did not increase the number of adverse drug events or infusion reactions, either after switching or related to the switch [32].

Study 4 was a switch from ADL (Humira) to the biosimilar SB5 in patients with IBD. A multicenter, phase IV, interventional cohort study was performed over 12 months. Patients were previously educated about biosimilars before switching from ADL (Humira) to SB5 to prevent the nocebo effect. Then the patients were able to switch, and a 75% persistence rate was observed after 12 months. Few patients discontinued switching because of adverse events caused by injection site pain within 30 min after the SB5 injection [33].

In Study 5 patients with IBD and rheumatic conditions switched from ADL (Humira) to the SB5. This was an observational 12-month study involving a multicenter cohort of patients who were naïve to ADL or who were already being treated with ADL. This study demonstrated that SB5 is clinically effective and has a safety profile similar to ADL, without increased adverse events or loss of efficacy. SB5 effectiveness was demonstrated for both patients who were naïve to ADL and patients who had switched [34].

### Psoriasis and PsA

Study 1 is a switch from ustekinumab (Stelara) to the biosimilar AVT04 in patients with moderate-to-severe chronic psoriasis. Psoriasis is a skin condition characterized by red, scaly patches that can cause pain, itching, and inflammation. Ustekinumab is a monoclonal antibody that targets IL-12 and IL-23, cytokines involved in the inflammatory process of psoriasis (Table 8). A multicenter, randomized, double-blind clinical trial was conducted over 52 weeks. Patients were randomly assigned to receive AVT04 or ustekinumab treatment in a blinded manner. This study demonstrated equivalence between AVT04 and ustekinumab, with similar safety and efficacy [35].

**Table 8:** Summary of psoriasis and PsA studies

Study	Description	Aim	Type of study	Outcome	References
1	Switching from ustekinumab to AVT04	Evaluate efficacy, safety, tolerability and immunogenicity	Multicenter, randomized, double-blind	Demonstrate equivalence between treatments with similar safety and efficacy	[35]
2	Switching from ADL to AVT02	Evaluate efficacy, safety and immunogenicity	Phase III, multicenter, randomized, double-blind, parallel group conducted in (Estonia, Georgia, Poland and Ukraine)	Demonstrated to be safe and well tolerated, with safety and immunogenicity equivalence. It was able to provide improvements on the quality of life of patients	[36]

Study 2 is a switch from ADL to the biosimilar AVT02 in patients with moderate-to-severe chronic psoriasis. A multicenter, randomized, double-blind, parallel-group phase III clinical trial was conducted in four countries (Estonia, Georgia, Poland, and Ukraine) over 52 weeks. AVT02 was demonstrated to be safe and well tolerated, with safety and immunogenicity equivalent to ADL, no clinical differences, and improved patients’ quality of life compared with the reference medicine [36].

### Switching Studies Applicable to Multiple IMIDs

Two clinical trials (Study 1) were performed on switching from reference ADL to biosimilars; the VOLTAIRE-X study was performed on switching to the ADL-adbm, and the REFLECTIONS B538-12 study was performed on switching to the biosimilar ADL-afzb. Both studies were phase III trials that included multiple switched-in patients with chronic psoriasis and RA (Table 9). Safety and pharmacokinetic equivalence were observed in both studies, allowing interchangeability [31].

**Table 9:** Summary of the switching studies applicable to multiple IMIDs

Study	Description	Aim	Type of study	Outcome	References
1	Switching from ADL to ADL-adbm and ADL-afzb	Evaluate the efficacy and safety	Phase III trial that included multiple switched in patients with chronic plaque psoriasis and rheumatoid arthritis	Safety and pharmacokinetic equivalence were observed	[31]
2	Switching from infliximab to CT-P13	Demonstrate equivalence	Randomized study in patients with axial spondyloarthritis and with rheumatoid arthritis	No clinically meaningful differences	[37]
3	Switching from infliximab to CT-P13	Demonstrate equivalence	Phase IV trial study, randomized, double-blind and non-inferiority in patients with axial spondyloarthritis, rheumatoid arthritis, Crohn’s disease and psoriasis	Patients under stable infliximab treatment did not achieve disease remission during long-term therapy	[38]
4	Switching from infliximab to CT-P13	Evaluate the efficacy, safety and immunogenicity	Phase III, non-inferiority, double-blind study was performed in biologic-naïve patients with Crohn’s disease	No differences in efficacy, safety, or immunogenicity were observed	[39]

The PLANETAS Study 2, a randomized study comparing switching from infliximab to CT-P13, conducted in 221 patients with axSpA, demonstrated equivalence over 30 weeks. Consistent with the PLANETAS study, the PLANETRAS study in 606 RA patients demonstrated comparable efficacy and safety at week 30 [37].

NOR-SWITCH was a phase IV trial that was randomized, double-blind, and noninferiority, conducted in 482 patients with different conditions (axSpA, Crohn’s disease, psoriasis, PsA, RA, and UC) on stable treatment, with infliximab switched to CT-P13 or continued on the reference medicine. This study demonstrated noninferiority in real-world populations and revealed that patients under stable infliximab treatment did not achieve disease remission during long-term therapy [38].

Study 3 is a phase III, noninferiority, double-blind study performed in biologic-naïve patients with Crohn’s disease to evaluate the equivalence between infliximab and the CT-P13 for 30 weeks. After week 30, all patients received CT-P13, and no differences in efficacy, safety, or immunogenicity were observed [39].

Considering the previously analyzed outcomes, all studies demonstrated equivalence in safety, efficacy, and immunogenicity between the biosimilar and the reference medicine when switching to a biosimilar in each IMID studied (RA, psoriasis, PsA, and IBD). However, variability in persistence and patient-reported outcomes highlights the critical influence of patient perception and expectations. Some studies have shown that educational interventions before switching are associated with greater treatment persistence and fewer discontinuations, strongly indicating that the nocebo effect is a key determinant of real-world biosimilar performance. For example, in the SB5 switching study for IBD, patients who were proactively informed about biosimilars before switching achieved a 75% persistence rate after 12 months, despite mild injection-site reactions [33]. Conversely, studies in which switching occurred without adequate communication frequently report higher rates of perceived loss of response or intolerance, even though objective disease activity remains unchanged [40]. Thus, real-world evidence in IMIDS supports a dual conclusion: biosimilars are clinically equivalent, but their success in practice depends on effective expectation management. Integrating nocebo-aware switching strategies into routine care is therefore as important as regulatory rigor in achieving sustainable biosimilar adoption [41].

Collectively, real-world and clinical trial evidence have demonstrated that biosimilars are clinically interchangeable with reference biologics across multiple IMIDs. No study has reported increased immunogenicity, loss of efficacy, or safety concerns following switching, including multiple or reverse switching. These data provide a strong scientific basis for regulatory decisions on extrapolation and interchangeability.

## Addressing Barriers to Biosimilar Adoption

Biosimilars adoption is often suboptimal due to barriers like limited healthcare professional knowledge and confidence, patient switching concerns, inconsistent policies, and lack of harmonized guidelines.

Physician hesitation is often driven by uncertainty regarding interchangeability, immunogenicity, and long-term safety, despite robust regulatory standards and growing real-world evidence. Similarly, clinical evidence indicates that discontinuations and reported adverse events are often driven by patient perception and nocebo responses rather than by true pharmacological differences [42]. Therefore, physicians play an important role in overcoming barriers to biosimilar adoption by educating patients about biosimilars, sharing their knowledge with colleagues, implementing strategies to reduce the nocebo effect, and adopting a joint decision-making approach [14, 29]. Collaboration among all stakeholders (patients, pharmacists, physicians, manufacturers, healthcare professionals, and systems) is fundamental to ensuring confidence in the adoption and use of biosimilars as intended, thereby generating market competition [29].

The EMA has established a strong educational and regulatory framework through its website, including guidance for healthcare professionals and patients, Q&A documents, and the European Public Assessment Reports (EPARs) [17]. However, these resources are not systematically integrated into national switching policies or clinical workflows, limiting their impact on real-world biosimilar adoption.

The EPAR provides a comprehensive regulatory record of each EMA-approved biosimilar, including detailed scientific evaluations of comparability, manufacturing, clinical studies, immunogenicity, and postauthorization safety. However, their technical complexity and limited accessibility for nonspecialists mean that EPARs are underused by clinicians and patients in routine decision-making, creating an implementation gap between regulatory evidence and clinical confidence. Although EPARs provide a high level of regulatory transparency, their limited visibility in routine clinical practice and lack of integration into prescribing systems represent a critical implementation gap that reduces their ability to support switching decisions and mitigate nocebo effects.

Table 10 highlights four interrelated strategies essential to overcoming key barriers to biosimilar adoption: physician–patient communication, multidisciplinary management, social observational learning, and monitoring and mitigation of the nocebo effect. Together, these strategies address not only scientific and regulatory concerns but also the behavioral, psychological, and organizational factors that influence biosimilar uptake in clinical practice. Physicians’ confidence in biosimilars strongly shapes patient perceptions. When clinicians express positive, evidence-based views about biosimilars and actively involve patients in treatment decisions, patients are more likely to feel empowered and to accept biosimilar therapies. Shared decision-making fosters trust and reduces uncertainty, which is particularly important during treatment transitions.

**Table 10:** Strategies to address confidence barriers and mitigate the nocebo effect in biosimilar use

Domain	Strategies
Physician–patient communication	Physicians’ positive and confident perception of biosimilars Active patient involvement in the decision-making process to promote empowerment
Multidisciplinary management	Team-based collaboration among gastroenterologists, nurse practitioners, physician assistants, nurses, and pharmacists to provide consistent patient education on biosimilars
Social observational learning	Consideration of individual patient characteristics (e.g., prior treatment history, educational level) to tailor education and facilitate shared decision-making
Monitoring and detection of the nocebo effect	Use of validated tools to identify patients’ expectations and those at increased risk of nocebo responses Examples include the Perceived Sensitivity to Medicines scale and the Stanford Expectations of Treatment Scale, which assess concerns, confidence, and preconceived beliefs about medication

The role of multidisciplinary management enhances communication. Biosimilar education and implementation benefit from collaboration among gastroenterologists, nurse practitioners, physician assistants, nurses, and pharmacists. Each offers unique expertise, from clinical decisions and patient counselling to medication management and adherence monitoring. This team-based approach ensures consistent messaging, reduces misinformation, and provides patients with chances to ask questions and address concerns, boosting confidence in biosimilar therapies.

Social observational learning influences biosimilar adoption by shaping patients’ beliefs through direct communication and personal experiences. Considering individual factors like treatment history, education, and health literacy helps healthcare providers customize education and communication strategies. When patients see peers with positive biosimilar outcomes, their own acceptance and confidence in these medicines are reinforced, facilitating wider use [43]. Monitoring and managing the nocebo effect is vital, as negative expectations can cause perceived inefficacy or more adverse reports, even without pharmacological differences. Using validated tools, such as scales for perceived sensitivity and treatment expectations, helps clinicians identify patients at higher risk of nocebo responses. Early detection allows for targeted interventions, such as counseling and reassurance, to mitigate negative perceptions and improve outcomes.

Successful biosimilar integration needs more than regulation and cost benefits. It requires a patient-centered approach with clear communication, coordinated healthcare, tailored education, and proactive management of expectations. Addressing these factors can boost biosimilar acceptance, improve outcomes, and maximize benefits [29].

Table 11 describes the main suggested strategies to consider for adoption to address the main barriers identified in the clinical practice of biosimilars in IMIDs. These initiatives would help healthcare providers and decision-makers become more aware of and educated about the economic benefits of biosimilar use and more confident in the safety and efficacy of biosimilars [24].

**Table 11:** Biosimilar barriers in IMIDs and strategies to maximize their use

Barriers identified	Possible strategies
Extrapolation of data	Transparency and availability of data from clinical trials
Nocebo effect	Introduction of a joint decision-making approach Promote educational initiatives and awareness on biosimilars, and explain the rationale for switching
Interchangeability	Promote medical associations’ initiatives to discuss and educate on interchangeability Introduce incentives to healthcare professionals
Regulation	Provide clear guidance on how to switch and manage the nocebo effect Harmonize the rationale to switch on a national level

## Conclusion and Future Perspectives

Biosimilars are a major advance for managing IMIDs within treat-to-target strategies, offering biologic-level effectiveness while reducing overall treatment costs and expanding earlier access to advanced therapies. Ensuring broad, long-term availability of these lower-cost options is essential, given the chronic and financially burdensome nature of IMIDs.

Extensive evidence confirms that biosimilars match reference biologics in safety, efficacy, and immunogenicity, yet their optimal use remains hindered by regulatory ambiguities and stakeholder uncertainty. Key gaps persist in switching practices, interchangeability, and management of the nocebo effect, compounded by the limited dissemination of randomized and real-world switching data.

Clearer, harmonized European regulatory guidance is needed—especially on switching protocols, interchangeability, and nocebo prevention strategies—along with improved access to EPARs and real-world evidence. Policies should align incentives with patient-centred care, supported by standardized communication, shared decision making, and structured follow-up after switching.

For both clinicians and patients, trust, education, and transparent communication are central to successful biosimilar adoption. Multidisciplinary teams, including clinical pharmacists, play a critical role in ensuring consistent, evidence-based guidance. Strengthened registries and pharmacovigilance should track safety, persistence, patient-reported outcomes, and switching-related discontinuations.

Ultimately, optimizing biosimilar use in IMIDs requires coordinated efforts across the regulatory, clinical, and patient-engagement domains. By addressing the nocebo effect, standardizing switching practices, and embedding shared decision-making into routine care, biosimilars can become not only cost-effective alternatives but also fully trusted components of sustainable, high-quality IMID management.

## Data Availability

All data used are available in the public domain of the references.

## References

[1] David T, Ling SF, Barton A. Genetics of immune-mediated inflammatory diseases. Clin Exp Immunol. 2018;193(1):3–12. 29328507 10.1111/cei.13101 PMC6037997

[2] El-Gabalawy H, Guenther LC, Bernstein CN. Epidemiology of immune-mediated inflammatory diseases: incidence, prevalence, natural history, and comorbidities. J Rheumatol Suppl. 2010;85:2–10. 20436161 10.3899/jrheum.091461

[3] Hjuler KF, Møller LF, Elgaard CDB, et al. On the interdisciplinary treatment and management of patients with immune-mediated inflammatory diseases. a study on patients' personal experiences and perspectives. J Multidiscip Healthc. 2024;17:2635–2646. 38828269 10.2147/JMDH.S432820 PMC11141566

[4] Liu C, Chu D, Kalantar-Zadeh K, et al. Cytokines: from clinical significance to quantification. Adv Sci (Weinh). 2021;8(15):e2004433. 34114369 10.1002/advs.202004433 PMC8336501

[5] McInnes IB, Gravallese EM. Immune-mediated inflammatory disease therapeutics: past, present and future. Nat Rev Immunol. 2021;21(10):680–686. 34518662 10.1038/s41577-021-00603-1 PMC8436867

[6] McLean AK, Reynolds G, Pratt AG. Leveraging multi-tissue, single-cell atlases as tools to elucidate shared mechanisms of immune-mediated inflammatory diseases. Biomedicines. 2024;12(6):1297. 38927506 10.3390/biomedicines12061297 PMC11201400

[7] Kuek A, Hazleman BL, Östör AJK. Immune-mediated inflammatory diseases (IMIDs) and biologic therapy: a medical revolution. Postgrad Med J. 2007;83(978):251–260. 17403952 10.1136/pgmj.2006.052688 PMC2600039

[8] Dinarello CA. Historical insights into cytokines. Eur J Immunol. 2007;37(Suppl 1):S34–S45. 17972343 10.1002/eji.200737772 PMC3140102

[9] Molinelli E, Campanati A, Ganzetti G, et al. Biologic therapy in immune mediated inflammatory disease: basic science and clinical concepts. Curr Drug Saf. 2016;11(1):35–43. 26463248 10.2174/1574886310666151014115127

[10] Kim H, Alten R, Avedano L, et al. The future of biosimilars: maximizing benefits across immune-mediated inflammatory diseases. Drugs. 2020;80(2):99–113. 32002851 10.1007/s40265-020-01256-5 PMC7007415

[11] Rezk MF, Pieper B. Unlocking the value of anti-TNF biosimilars: reducing disease burden and improving outcomes in chronic immune-mediated inflammatory diseases: a narrative review. Adv Ther. 2020;37(9):3732–3745. 32740789 10.1007/s12325-020-01437-4 PMC7444394

[12] Barbier L, Simoens S, Vulto AG, et al. European stakeholder learnings regarding biosimilars: part II-improving biosimilar use in clinical practice. BioDrugs. 2020;34(6):797–808. 33063267 10.1007/s40259-020-00440-z PMC7669768

[13] Ingrasciotta Y, Cutroneo PM, Marcianò I, et al. Safety of biologics, including biosimilars: perspectives on current status and future direction. Drug Saf. 2018;41(11):1013–1022. 29796832 10.1007/s40264-018-0684-9

[14] Edwards CJ, Hercogová J, Albrand H, et al. Switching to biosimilars: current perspectives in immune-mediated inflammatory diseases. Expert Opin Biol Ther. 2019;19(10):1001–1014. 31056970 10.1080/14712598.2019.1610381

[15] Fiorino G, Girolomoni G, Lapadula G, et al. The use of biosimilars in immune-mediated disease: a joint Italian Society of Rheumatology (SIR), Italian Society of Dermatology (SIDeMaST), and Italian Group of Inflammatory Bowel Disease (IG-IBD) position paper. Autoimmun Rev. 2014;13(7):751–755. 24657898 10.1016/j.autrev.2014.02.004

[16] Krämer I, Thiesen J, Astier A. Formulation and administration of biological medicinal products". Pharm Res. 2020;37(8):159. 32743712 10.1007/s11095-020-02859-z

[17] European Medicines Agency. Biosimilars in the EU—information guide for healthcare professionals [Internet]. 2023 Nov 13 [cited 2024 Oct 20]. Available from: https://www.ema.europa.eu/en/documents/leaflet/biosimilars-eu-information-guide-healthcare-professionals_en.pdf.

[18] Cordeiro MA, Vitorino C, Sinogas C, et al. A regulatory perspective on biosimilar medicines. Pharmaceutics. 2024;16(3):321. 38543215 10.3390/pharmaceutics16030321 PMC10974016

[19] Ho K. Manufacturing process of biologics. International conference on harmonisation of technical requirements for registration of pharmaceuticals for human use. 2011 [cited 15 Nov 2024]. Available from: https://www.ema.europa.eu/en/documents/presentation/presentation-manufacturing-process-biologics-kowid-ho-afssaps_en.pdf

[20] ICH Q11 Development and manufacture of drug substances (chemical entities and biotechnological/biological entities) - scientific guideline. 2012 [cited 16 Nov 2024]. Available from: https://www.ema.europa.eu/en/ich-q11-development-manufacture-drug-substances-chemical-entities-biotechnological-biological-entities-scientific-guideline

[21] Rugo HS, Rifkin RM, Declerck P, et al. Demystifying biosimilars: development, regulation and clinical use. Future Oncol. 2019;15(7):777–790. 30500264 10.2217/fon-2018-0680

[22] Rezk MF, Pieper B. Treatment outcomes with biosimilars: be aware of the nocebo effect. Rheumatol Ther. 2017;4(2):209–218. 29032452 10.1007/s40744-017-0085-z PMC5696297

[23] Mascarenhas-Melo F, Diaz M, Gonçalves MBS, et al. An overview of biosimilars-development, quality, regulatory issues, and management in healthcare. Pharmaceuticals (Basel). 2024;17(2):235. 38399450 10.3390/ph17020235 PMC10892806

[24] Clarke K, Ainslie-Garcia M, Ferko N, et al. Modelling the opportunity for cost-savings or patient access with biosimilar adalimumab and tocilizumab: a European perspective. J Med Econ. 2024;27(1):952–962. 39015093 10.1080/13696998.2024.2379212

[25] Fleischmann RM, Alvarez DF, Bock AE, et al. Long-term efficacy, safety, and immunogenicity of the adalimumab biosimilar, PF-06410293, in patients with rheumatoid arthritis after switching from reference adalimumab (Humira®) or continuing biosimilar therapy: week 52-92 data from a randomized, double-blind, phase 3 trial. Arthritis Res Ther. 2021;23(1):248. 34563243 10.1186/s13075-021-02626-4 PMC8464121

[26] Shim SC, Božić-Majstorović L, Berrocal Kasay A, et al. Efficacy and safety of switching from rituximab to biosimilar CT-P10 in rheumatoid arthritis: 72-week data from a randomized Phase 3 trial. Rheumatology (Oxford). 2019;58(12):2193–2202. 31184752 10.1093/rheumatology/kez152 PMC6880852

[27] Blauvelt A, Leonardi CL, Gaylis N, et al. Treatment with SDZ-ADL, an adalimumab biosimilar, in patients with rheumatoid arthritis, psoriasis, or psoriatic arthritis: results of patient-reported outcome measures from two phase III studies (ADMYRA and ADACCESS). BioDrugs. 2021;35(2):229–238. 33651341 10.1007/s40259-021-00470-1 PMC7952364

[28] Schreiber S, Puig L, Gonçalves J, et al. Critical appraisal and future outlook on anti-inflammatory biosimilar use in chronic immune-mediated inflammatory diseases. Semin Arthritis Rheum. 2022;55:152023. 35640490 10.1016/j.semarthrit.2022.152023

[29] Angyal A, Bhat S. Biosimilars in IBD: what every clinician needs to know. Curr Gastroenterol Rep. 2024;26(3):77–85. 38243154 10.1007/s11894-023-00913-5

[30] Jorgensen KK, Olsen IC, Goll GL, et al. Switching from originator infliximab to biosimilar CT-P13 compared with maintained treatment with originator infliximab (NOR-SWITCH): a 52-week, randomised, double-blind, non-inferiority trial. Lancet. 2017;389:2304–2316. 28502609 10.1016/S0140-6736(17)30068-5

[31] Hanauer S, Liedert B, Balser S, et al. Safety and efficacy of BI 695501 versus adalimumab reference product in patients with advanced Crohn's disease (VOLTAIRE-CD): a multicentre, randomised, double-blind, phase 3 trial. Lancet Gastroenterol Hepatol. 2021;6(10):816–825. 34388360 10.1016/S2468-1253(21)00252-1

[32] Haifer C, Srinivasan A, An YK, et al. Switching Australian patients with moderate to severe inflammatory bowel disease from originator to biosimilar infliximab: a multicentre, parallel cohort study. Med J Aust. 2021;214(3):128–133. 33070332 10.5694/mja2.50824

[33] Deprez N, De Somer T, Baert D, et al. Evaluation of the safety and effectiveness after switch from adalimumab originator to biosimilar SB5 in patients with inflammatory bowel disease in a real-life setting. Acta Gastroenterol Belg. 2022;85(4):557–564. 36566364 10.51821/85.4.10724

[34] Fautrel B, Bouhnik Y, Salliot C, et al. Real-world evidence of clinical outcomes of the use of the adalimumab biosimilar SB5 in rheumatic and gastrointestinal immune-mediated inflammatory diseases: 12-month data from the PERFUSE study. Drugs Real World Outcomes. 2024;11(4):573–591. 39384685 10.1007/s40801-024-00459-6 PMC11589083

[35] Feldman SR, Reznichenko N, Berti F, et al. Randomized, double-blind, multicenter study to evaluate efficacy, safety, tolerability, and immunogenicity between AVT04 and the reference product ustekinumab in patients with moderate-to-severe chronic plaque psoriasis. Expert Opin Biol Ther. 2023;23(8):759–771. 37435850 10.1080/14712598.2023.2235263

[36] Feldman SR, Reznichenko N, Pulka G, et al. Efficacy, safety and immunogenicity of AVT02 versus originator adalimumab in subjects with moderate to severe chronic plaque psoriasis: a multicentre, double-blind, randomised, parallel group, active control, phase III study. BioDrugs. 2021;35(6):735–748. 34657274 10.1007/s40259-021-00502-w PMC8520467

[37] Park W, Hrycaj P, Jeka S, et al. A randomised, double-blind, multicentre, parallel-group, prospective study comparing the pharmacokinetics, safety, and efficacy of CT-P13 and innovator infliximab in patients with ankylosing spondylitis: the PLANETAS study. Ann Rheum Dis 2013;72(10):1605–12. 23687259 10.1136/annrheumdis-2012-203091 PMC3786643

[38] Goll GL, Jørgensen KK, Sexton J, et al. Long-term efficacy and safety of biosimilar infliximab (CT-P13) after switching from originator infliximab: open-label extension of the NOR-SWITCH trial. J Intern Med. 2019;285(6):653–669. 30762274 10.1111/joim.12880 PMC6850326

[39] Ye BD, Pesegova M, Alexeeva O, et al. Efficacy and safety of biosimilar CT-P13 compared with originator infliximab in patients with active Crohn's disease: an international, randomised, double-blind, phase 3 non-inferiority study. Lancet 2019;393(10182):1699–1707. 30929895 10.1016/S0140-6736(18)32196-2

[40] Cohen HP, Hachaichi S, Bodenmueller W, et al. Switching from one biosimilar to another biosimilar of the same reference biologic: a systematic review of studies. BioDrugs. 2022;36(5):625–637. 35881304 10.1007/s40259-022-00546-6 PMC9485085

[41] Fleischmann R, Jairath V, Mysler E, et al. Nonmedical Switching From Originators to Biosimilars: Does the Nocebo Effect Explain Treatment Failures and Adverse Events in Rheumatology and Gastroenterology Rheumatol Ther 2020;7(1):35–64. 31950442 10.1007/s40744-019-00190-7 PMC7021884

[42] Barbier L, Simoens S, Vulto AG, et al. European stakeholder learnings regarding biosimilars: part I-improving biosimilar understanding and adoption. BioDrugs. 2020;34(6):783–796. 33141421 10.1007/s40259-020-00452-9 PMC7669769

[43] Feagan BG, Marabani M, Wu JJ, et al. The challenges of switching therapies in an evolving multiple biosimilars landscape: a narrative review of current evidence. Adv Ther. 2020;37(11):4491–4518. 32910420 10.1007/s12325-020-01472-1 PMC7547992

